# 

**Published:** 1803-06-01

**Authors:** 


### To CORRESPONDENTS.

We have no other motive for postponing Dr. Hume's case, but the conviction that its publication at present could answer no useful purpose.

Communications are received from Dr. Mossman, Mess. Giraud, Hickin, Simmons, Robertson, N. G. Iatrice, Tinicola, and X.

We wish to remind our Correspondents that anonymous cases are not considererd by the public as medical evidence.

END OF VOL. IX.

---

[ W. Thorne, Printer, Red Lion Court, Fleet Street. ]

### INDEX.

ABERNETHY's, Mr. account of a new gorget for lithotomy, *(with a plate)* 394, 566
Acetite of lead, obfervations on 173
Acharius, M. on the ufe and advantages of tar water in venereal complaints 581
Acid, obfervations on the febacic 78
——, on the virtues of the phofphoric 388
—— phofphat of lime, characters of 493
—— of the uric 491
Adams, Dr. on morbid poifons 309
Adhefions, on thoracic 359
Adipocire, characters of the 497
Æther, on the effects of phofphorated 300
Affufion, the effects of cold, in typhus 9, 206
——, on the benefits of, in gout, 116, 124, 205, 362, 442
Air-pump-vapour bath, benefits of the 175
Alchol, obfervations on 40
Aldini, prof. his experiments on the body of a malefactor 195, 382
——, remarks on the experiments of 446
Alkali, on the virtues of fixed 176
Allen, Mr. his cafe of phthifis 263
Amaurofis, method of applying galvanifm in 131
——, cafes of 138
America, account of the peftilential fever in 110
——, fuccefs of vaccine inoculation in 196
Ammoniaco-magnefian phofphat, characters of the 494
Amputation of the thigh, cafe of 499
Anatomy, the ftudy of, recommended in education 477
Aneurifm, obfervations on 88, 180
Anger, remarkable effects of 288
Angerftein's, Mr. letter to Lord Hardwicke 540
Angina maligna, on the treatment of 249
Animal bezoardic refin, characters of 498
—— concretions on the materials which compofe different 489
—— body, on its analogy with vegetable organization 583
Animation, on the reftoration of fufpended 291
Apology for differing from the Monthly and Critical Reviewers, Dr. Lettfom's 90
Apoplexy, Dr. Magennis's cafes and obfervations on 320
——, Dr. Moffman on 409
Apparent death, caution refpecting 73
Aqua laurocerafi, mode of preparing 288
Ariftotle's opinion concerning hydrophobia 390
Armiger's, Mr. medical teaching announced 96
Aromatic vinegar, Mr. Henry's claim to the difcovery of 307
——, Dr. Trotter on 448
Arthritis, Dr. Kinglake on cold affufion in 116, 442
——, Mr. Cuftance on 124
——, Mr. Wadd's Remarks on 205
——, a conftant reader on 362
Afcites, cafe of 211
Afia, progrefs of vaccine inoculation in 450
Auditory organ, benefit of galvanifm in difeafes of the 133
Aurum paradoxum, analysis of the 473
Autumnal fever, obfervations on 213
Bagdad, fuccefs of the vaccine innoculation at 451
Ball-forceps, account of a new 2
Bamberg, on the treatment of difeafes in the hofpital at 268
Bandage, obfervations on the plafter 113
Bardfley, Dr. on influenza 522
Barlow's, Mr. cafe of premature delivery 403
Bafaltes, chemical analyfis of the 475
Bath, on the air-pump vapour 175
Batty's, Dr. Lectures announced 96, 292, 584
Beddoes, Dr. on phthifis 264
——, on the influenza 460
Bell's, Mr. principles of furgery reviewed 84, 177, 276
Berlinghieri, Dr. account of a new work of 437
Berthollet, cit. on the comparative effects of light and heat 83
Beryll, chemical analysis of the 475
*Beytrage zur chemifchen kentnifs der mineral korper*, reviewed 472
Bezoards, on the materials of 489
Birch, Mr. on medical electricity, reviewed 286
Bitumen, analyfis of the elaftic 447
Bleeding, its ufe in the yellow fever 581
Blegborough's Dr. cafe of paralyfis 175
—— facts and obfervations refpecting the air-pump vapour bath, reviewed 482
Blizard's Mr. lectures announced 96
Blood, Galvanic experiment on the 291
Bockmann, prof. on the putrefaction of flefh in gas 219
BOOKS, MEDICAL, CRITICAL ANALYSIS OF—The Principles of Surgery, in two volumes: Volume first, of the Ordinary Duties of the Surgeon, &c. Volume fecond, a Syftem of Surgical Operations, &c. by John Bell, 84, 177, 276. Effays on the Difeafes of Children, with Cafes and Diffections, by John Cheyne, M.D. 89. An

Apology for differing in Opinion from the Authors of the Monthly and Critical Reviews, on the Subject of Variolous and Vaccine Inoculation, &c. by J. C. Lettfom, M. D. and LL. D. 90. A collection of papers intended to promote an Inftitution for the Cure and Prevention of infectious Fevers in Newcaftle and other populous towns, by John Clark, M. D. 182. A Letter to Sir Walter Farquhar, Bart. on the Subject of a particular Affection of the Bowels, very frequent and fatal in the Eaft Indies, by Fra. Duncan, 183. Medicina Nautica, an Effay on the Difeafes of Seamen, vol. 3, by Thomas Trotter, M. D. 185, 282. Obfervations on the Conftitution of Women, and on fome of the Difeafes to which they are more efpecially liable, by Sayer Walker, M. D. 279. Extract from an Account of Cafes of Typhus Fever, in which the Affufion of cold Water has been applied in the London Houfe of Recovery, by W. P. Dimfdale, M. D. 282. A new Medical Dictionary, containing a concife Explanation of all the Terms ufed in Medicine, Surgery, Pharmacy, Botany, Natural Hiftory, and Chemiftry, by Jofeph Fox, M. D. and Thomas Bradley, M. D. 285. Obfervations on Dr. Pearfon's Examination of the Report of the Vaccine Committee of the Houfe of Commons concerning Dr. Jenner's Claim for Remuneration, by Thomas Creafer, 286. An Effay on the Medical Application of Electricity, by John Birch, Efq. *ib.* An Account of the Difcovery and Operation of a new Medicine for Gout, *ib.* Some Obfervations on the prefent Epidemic Catarrhal Fever or Influenza, chiefly in relation to its Mode of Treatment, by Richard Pearfon, M. D. 377. Cafes of Cancer with Obfervations on the ufe of Carbonate of Lime in that Difeafe, by Edward Kentifh, M. D. 380. An Account of the Galvanic Experiments performed by John Aldini, Profeffor of Experimental Philofophy in the Univerfity of Bologna, on the Body of a Malefactor executed at Newgate, 382. Obfervations on the Epidemical Difeafes now prevailing in London, by Robert Hooper, M. D. 385. *Beytrage zur Chemifchen Kentnifs der Mineral Korper*; i. e. Contributions to the Chemical Knowledge of Mineral Bodies, by M. H. Klaproth, 472. A popular View of the Structure and Economy of the Human Body, &c. by John Feltham, 476. Obfervations on a late Publication of Dr. Pearfon, entitled, "An Examination of the Report of the Committee of the Houfe of Commons, &c. by Henry Hicks, 481. On the Inutility and Dangers of Vaccine Inoculation, proved by Facts, by Cit. Goetz, of Paris, 482. Facts and Obfervations refpecting the Air-Pump-Vapour-Bath, *ib.* The Domeftic Medical Guide, by R. Reece, 485. An Inquiry into fome of the Effects of the Venereal Poifon on the Human Body, by R. Sawrey, 571. Attempt to inveftigate the Caufe of the Egyptian Ophthalmia, by G. Power, 575

Books, new medical announced. —Dr. Currie's new Edition of Medical Reports, 8. Dr. Trotter's third Volume of Medicina Nautica, 96. Dr. Walker's Obfervations on the Conftitution of Women, *ib.* A Tranflation of Sue's Hiftory of Galvanifm, 196. Mr. Power's Obfervations on the Ophthalmia of Egypt, *ib.* Mr. Wilkinfon, on the broad Leaved Willow Bark, 392. Dr. Jamefon's Enquiry into the Effects of Climate upon the Human Body, *ib.* Mr. Cooper's Treatife on the Anatomy and Surgical Treatment of Hernia, 488. Mr. Wilkinfon's Hiftory of Galvanifm, *ib.*

Boracites, new analyfis of the 92

Borrett's, Mr. case of amputation of the thigh 499

Bofquillon, M. on hydrophobia 390

Boftock's, Mr. obfervations on the urine 349

Bougies, advantages of cauftic 190

Bouttatz's, Dr. travels through Ruffia to extend vaccine inoculation 584

Bowels, on a particular affection of the 183

Boy, extraordinary effects of paffion in a 288

Bradley's, Dr. Medical Dictionary, reviewed 285

Brain, on concuffion of the 197

Brera, Dr. account of a new work by 437

Breflaw, progrefs of vaccine inoculation at 128

Brugnatelli, Signor, account of a new work of 436

Brunonian fyftem, fallacy of the 339

——————, its progrefs in Italy 434

Bubonocele, Mr. Cooper on the radical cure of the 161, 367

—————— Mr. Carlifle on the 396

Buchholz, Mr. on the preparation of the mild fublimate of mercury 81

——————, on the chemical analyfis of opium 398

Burke, Dr. on the yellow fever 37

Buffora, fuccefs of vaccine inoculation at 450

Caldwell, Dr. on the vitality of the blood, &c. 581

Cæfarean operation, obfervations on the 76

Calculi, on the urinary in men 242
——— account of extraordinary 268
———, on the materials which compofe 489
Caldani, account of his anatomical work and experiments 437
Cancer, Dr. Kentifh's treatife on 381
Carbonat of lime, on its ufe in cancer 496
Carlisle, Mr. on the radical cure of bubonocele 396
Cataract, obfervations on 172
———, cafe of 198
Catarrhal fever, on the prefent epidemic 375
———, obfervations on the 377
Ceruffa acetata, obfervations on 172
Ceylon, fuccefs of vaccine inoculation at 457
Chaubry, M. on the effects of phofphorated ether 300
Chauffier, Cit. on the umbilico-mefenteric veffels 170
Chemical and medical experiments on the diabetes fauar 12
——— analyfis of opium 398
Chenevix's, Mr. analyfis of the humours of the eye 196
Chenopodium Ambrofiodes, ufes of the 94
Chevalier's, Mr. lectures announced 96
Cheyne's, Dr. effays on the difeafes of children, reviewed 89
Child, cafe of one born without limbs 63
Child-murder, obfervations on 74
Children, on the difeafes of 89
Chifholm, Dr. firft employed mercury in the yellow fever 101
Chlorofis, obfervations on 280
Chorea fancti viti, cafe of 468
Chriftie, Mr. on the fuccefs of vaccine inoculation in the eaft 457, 537
Clarke's, Dr. Thomas, account of new furgical inftruments *(with a plate)* 1
———, Dr. John, collection of papers on the cure and prevention of fever, reviewed 182
Cold, account of an extraordinary one at Paris 287
Cold affufion, on the ufe of, in gout, 116, 124, 205, 362, 442
———, its benefits in typhus 206
Concretions, on the materials of animal 489
Concuffion, cafe of 272
Confumption, on the virtues of the lichen-iflandicus in 20, 188
———, on the treatment of pulmonary 270
Contagion, obfervations on 218
Contractions, experiments on Galvanic 146, 235
Contributions to the chemical knowledge of mineral bodies, reviewed 472
Cooper, Mr. on the radical cure of bubonocele, 367
———, A. treatife on hernia announced 488
Copenhagen, on the vaccine report of 463
Copper, analyfis of the muriated and phofphorated 475
Corn, difcovery of phofphat of lime in feveral kinds of 583
Correfpondents, addreffes to 47, 196, 292, 392, 488, 584
Countenance, verfes to the human 480
Cow-pock matter, analyfis of, and experiments on the 67
———, directions for preferving 298, 397
———, Dr. Marcet on the prefervation of 464
Cow-pox, Mr. Maurice on the 38
———, Mr. Cuff on 66
———, report of the Finfbury Difpenfary on 274
———, Dr. Adams on the 309
———, Mr. Wagftaffe's cafes of 361
———, Mr. Wafhbourne's cafe of 370
———, on the origin of the 451
———, Mr. Chriftie on the 457, 537
Creafer's, Mr. obfervations on Dr. Pearfon's examination of the vaccine report, reviewed 286
Croup, on the caufes of 90
Cruikfhank, Mr. on his analyfis of urine 351
Cucumbers, on the virtues of the juice of 24
Cuff's, Mr. defence of vaccination 66
Currie's Dr. J. medical reports announced 8
———, Dr. W. on the treatment of the yellow fever 97
Cuftance, Mr. on cold water in the gout 124
Cutaneous complaints, oil of the walnut kernel recommended in 94
Cuthbertfon, Mr. account of his Galvanic apparatus 291
Cynanche trachealis, obfervation on 89
Darwin, Dr. on influenza 505
Davies's, Mr. cafe of tinea capitis 60
Davis, Dr. Mr. Cuff's reply to 66
Deafnefs, Galvanic influence in 290
De Carro, Dr. his letter to Dr. Jenner on vaccination 450
Delivery, cafe of premature 403
Demerara, progrefs of vaccine inoculation at 65
Dennett, Mr. on vaccination 363
———, on the influenza 366
Des Fontaines, Dr. his cafe of difeafed liver 126
Diabetes fauar, experiments on the 12
Difficulty of afcertaining the virtues of medicines 19
———, of fwallowing, obfervation on 293
Digitalis purpurea, its fuccefs in hydrops pectoris 44
———, and in phthifis 263
Dijon, account of the meeting of the academy at 371

Dimsdale, Dr. on cold affusion in typhus, 206, 282

Diseases, on the coincidence of 38

———, on concealed 78

———, on the treatment of several 268

———, of children, essays on reviewed 89

———, account of in an eastern district of London, from Nov. 20 to Dec. 2 , 1802 16

———, from Dec. 20, 1802, to Jan. 20, 1803 191

———, from Jan. 20 to Feb. 20 275

———, from Feb. 20 to March 20 375

———, from March 20 to April 20 469

———, from April 20 to May 20 570

Dislocations, on supposed 202

Domestic Medical Guide, review of the 485

Douglass, Mr. his method of treating the yellow fever 581

Dow, Dr. his case of diseased kidnies 438

Dropsies, on the treatment of 270

Dubois, M. on the use of moxas in loss of speech 389

Duncan, Mr. on a particular affection of the bowels in the East Indies, reviewed 183

Dutrouil, Cit. on the sensibility of plants 291

Dysphagia, observations on 293

Ear, method of applying Galvanism to the 134

——, Mr. Low's method of taking casts of the 551

Earnest, Mr. his case of ascites 211

East Indies, on a particular affection of the bowels in the 183

———, on the subject of the plague in the 502

Egypt, on the climate and diseases of 113

Egyptian natron, chemical analysis of the 474

Egyptian ophthalmia, attempt to investigate the cause of the 575

Electricity, on the medical application of 286

Electricity, cases of white swelling cured by 569

———, comparative effects between Galvanism and 291

———, case of chorea sancti viti, cured by 468

———, the same fluid as Galvanism 469

Elephantiasis, remarkable case of an 289

———, Mr. Ward s case of 545

Emerald, chemical analysis of the 475

Epidemical diseases prevalent in London, observations on the 385

Epidemic fever, observations on the 377

Epidemic scarlet fever, on an 157

Epidemic catarrhal fever, Dr. Woodforde on the 505

Epidemic catarrhal fever, Mr. Goodwin on the 509

———, Mr. Harrup on the 512

———, Mr. Wadley on the 515

———, Dr. Kinglake on the 517

———, Dr. Bardsley on the 522

Erdmann's, Dr. relation of the extraordinary effects of passion in a boy 288

Erysipelas, observations on cases of 13

Essay on the medical application of electricity reviewed 286

Essays on the diseases of children, reviewed 89

Ether, on the effects of phosphorated 300

Exeter, on the epidemic at, in 1729 505

Experiments, on mercurial salts 9

———, on the diabetes sauar 12

———, on alcohol 44

———, on the cow pock matter 67

———, on the application of Galvanism in the cure of diseases, by Dr. Grapengiesser 131

———, on Galvanic contractions, by Mr. Tupper 146, 235

———, by Prof. Aldini on the body of a malefactor 195, 382

———, on the humours of the eye 196

———, on the putrefaction of flesh in different kinds of gas 219

———, in Galvanism 290

———, relative to the chemical analysis of opium 398

Extenuation in the leg, case of a 231

Extract from an account of cases of typhus fever reviewed 282

Eye, analysis of the humours of the 196

——, on the extirpation of the globe of the *(with a plate)* 200

Facts and observations respecting the air-pump-vapour-bath, reviewed 484

Farquhar, Sir Walter, Mr. Duncan's letter to, on an affection of the bowels in the East Indies 183

———, Mr. Christie's letter to, on the success of vaccine inoculation, at Ceylon 459

Feltham's, Mr. popular view of the structure and œconomy of the human body, reviewed 476

Fever, on an epidemic scarlet 157

——, on the cure and prevention of 182

——, cases of typhus 206

——, on the epidemic catarrhal 377

——, see INFLUENZA

Fevers, method of treating intermittent 304, 567

———, on typhus 502

Fever, yellow, method of successfully treating 581

Finſbury Diſpenſary, ſurgical caſes admitted into the 13, 188, 272, 373, 467, 568
——, report on the vaccine inoculation 274
Fleſh, on the putrefaction of 219
Fœtus, memoir on the nutrition of the 255
——, on the particular conformation of a 461
Forceps, account of a newly invented ball 2
Foſſa navicularis, account of extraordinary calculi extracted from the 268
Fourcroy, Cit. his experiments on mercurial ſalts 9
——, on the urinary ſtones and calculi in men 242
——, on the number, nature, and characters of calculi, bezoards, and animal concretions *(with engravings)* 489
Fox's, Dr. Medical Dictionary reviewed 285
Fracture, caſe of 273
Frampton's, Mr. lectures announced 96
Freeman, Mr. on an epidemic ſcarlet fever and ſore throat 157
Frieſe, Dr. on vaccine inoculation 128
Fritze, Dr. on the virtues of the Iceland moſs 20
Frogs, Galvanic experiments on 487

Gadolinit, chemical analyſis of the 474

Gallini, Sig. his compendium of phyſiology and pathology noticed 437

Galvanic experiments, Profeſſor Aldini's 195, 382

—— contractions, experiments on 146, 235

—— apparatus, account of Mr. Pepys's 390

—— inſtrument, account of Mr. Cuthbertſon's 291

—— influence in deafneſs 290

—— ſtimulus, how to be applied in ſuſpended animation 291

—— influence upon the blood *ib.*

—— ſociety at Paris, report of the 487

Galvaniſm employed in the cure of ſeveral diſeaſes 131

——, tranſlation of Sue's hiſtory of announced 196

——, compared with electricity 291

——, important and ſtriking experiments in 290

——, announcement of a new hiſtory of 488

Gas, on the putrefaction of fleſh in different kinds of 219

Gelatine, obſervations on 499

Geſtation, ſingular caſe of ovarian 56

Gilbert, Mr. on digitalis in ſcarlatina 249

Giraud's, Mr. mode of preſerving vaccine matter 397

Glamorganſhire, laver bread eaten by the inhabitants of 24

Glaſs, Dr. on the epidemic at Exeter 505

Goetz, Cit. on the inutility of vaccine inoculation reviewed 484

Gonorrhœa, uſe of acetite of zinc in 53

Goodwin, Mr. on the prevailing epidemics 509

Gorget, deſcription of an improved *(with a plate)* 393, 566

Gout, Dr. Kinglake on the virtues of cold water in 116, 442

——, Mr. Cuſtance on the ſame 124

——, Mr. Wadd, on ditto 205

——, a conſtant reader on ditto 362

——, Meſſrs. Scott and Taynton ditto 547

——, Account of the diſcovery of a new medicine for, reviewed 286

Grapengieſſer, Dr. on the uſe of Galvaniſm in the cure of ſeveral diſeaſes 131

Guedeville's, Dr. experiments on the diabetes ſauar 12

Gunſhot wound, caſe of 165

Hæmorrhagy, Mr. Bell's obſervations on 87

Hæmorrhagies, uſe of the phoſphoric acid in 388

Hall's, Dr. obſervations on a French work reſpecting legal medicine 69

Hardwicke, Lord, his letter to J. J. Angerſtein, Eſq. on the vaccine inoculation 540

——, Dr. Jenner and Mr. Ring's anſwer to 541

Harris, Dr. on the malignant fever in the Weſt Indies 25

Harrup, Mr. on influenza 512

Headington's Mr. lectures announced 96

Heart, deſcription of the 478

Heat, on the comparative effects of light and 83

Heberden, Dr. correction of his evidence before the vaccine committee 95

Henning's, Dr. caſe of elephantiaſis 289

——, on the uſe of phoſphoric acid in hæmorrhagies 389

Henry, Mr. on the treatment of gonorrhœa 53

——, on the diſcovery of aromatic vinegar 307

——, Dr. Trotter's reply to 448

Henſler, Prof. on the leproſy 554

Hermaphrodite, account of 71

Hernia, on ſtrangulated ſcrotal 260

——, Mr. Cooper's treatiſe on announced 438

Hicks's, Mr. obſervations on a late publication of Dr. Pearſon's, reviewed 481

Hill's, Mr. remarks on the modus operandi of opium 153

Honey ſtone, chemical analyſis of the 474

Hooper, Dr. on the epidemical diſeaſes now prevailing in London, reviewed 385

Hooping cough, on the uſe of fixed alkali in 176

Hufeland, Dr. on the effects of the oil of walnuts in cutaneous complaints 54

Human countenance, verſes to the 480

Hunoldt's, Dr. analyſis and experiments on the cow-pock matter 67

Hunter, Mr. on ceruſſa acetata 173

Hutchins's, Mr. remarks on Galvaniſm 445

Hutchinſon's, Mr. caſe of ſteatomatous tumour *(with a plate)* 17

Hydrophobia, memoirs on 390

———, Ariſtotle's opinion of *ib.*

Hydrops pectoris, effects of digitalis in 94

Hydrothorax, caſe of 417

Iceland moſs, its virtues in phthiſis 20

Impey, Mr. on the uſe of vitriolated zinc 55

Infanticide, obſervations on 449

Inflammation in the maxillary ſinus, caſe of 423

Influenza, deſcription of the 375

———, Mr. Dennet on the 367

———, Dr. Pearſon's obſervations on, reviewed 377

———, Dr. Hooper's obſervations on, reviewed 385

———, Dr. Beddoes on the 460

———, Dr. Woodforde on the 505

———, Mr. Goodwin on the 509

———, Mr. Harrup on the 512

———, Mr. Wadley on the 515

———, Dr. Kinglake on the 517

———, Dr. Bardſley on the 522

———, Mr. Tomlinſon on the 530 *(note)*

Inſtruments, account of new ſurgical 1

Intermittent fevers, method of treating 305

Italy, ſtate of medical and phyſical literature in 435

Jail Fever, obſervations on 502

Jameſon's, Dr. treatiſe on the effects of climate on the human body announced 392

Jenner, Dr. correction of a miſtake in the memoirs of 62

———, vindication of 66

———, letter from Dr. De Carro to 450

———, correſpondence between Dr. Marcet and 462

———, his letter to Lord Hardwicke 541

Jennerian inſtitution, proceedings of the 102

———, patronized by their majeſties 292

Job, on the diſeaſes of 564

Joints, caſes of diſeaſed 374

Kentiſh's, Dr. caſe of cancer, with obſervations on the uſe of carbonat of lime in that diſeaſe, reviewed 380

Keyt's, capt. letter on vaccine inoculation in the iſland of Ceylon 391

Kidnies, caſe of diſeaſed 438

King's, Mr. caſe of gun-ſhot wound 165

———, caſe of inflammation in the maxillary ſinus 423

Kinglake, Dr. on cold applications in gout 116

Kinglake, Dr. vindication of ditto 442

——— on the influenza 517

Klaproth, M. H. *beytrage zur chemischen kentniſs der mineral korper*, reviewed 472

Knee-joints relieved by electricity 568

Klingſtone, chemical analyſis of the 475

Kryolithos, chemical analyſis of the *ib.*

Larrey's, Cit. account of a leech ſwallowed and ſtopped in the throat 262

Laudanum, its uſe in the yellow fever 100

Laurocerasi, how to prepare the aqua 288

Lead ores, chemical analyſis of 474-5

LECTURES ANNOUNCED.—Dr. Batty's on the theory and practice of midwifery, and on the diſeaſes of women and children, 96, 292, 584. Mr. Macartney on comparative anatomy and phyſiology, and on the ſtructure of vegetables, 96. Meſſrs. Headington and Frampton on anatomy, phyſiology, and the principles and operations of ſurgery, *ib.* Meſſrs. Blizards on ſurgery, *ib.* Mr. Chevalier on the principles and operations of ſurgery, *ib.* Meſſrs. Wagſtaffe and Syer on the elements and practice of Midwifery, *ib.* 292. Mr. Thomas on the practice and operations of ſurgery 196

Leech, account of a peculiar one ſwallowed and ſtopped in the throat 262

Leg, ſingular extenuation of the 231

Lentin's Dr. two caſes of hydrops pectoris 94

Leproſy, on that of the middle ages 554

Leſte, account of a wind ſo called in Madeira 315

Lettſom's, Dr. apology for differing from the Monthly and Critical Reviews, account of 90

Lichen Iſlandicus, on the virtues of 20

———, where to be procured 292

———, its virtues in phthiſis 488

Light and heat, on the comparative effects of 83

Limb, caſe of an uncommon affection in a 233

Limbs, caſe of a living child born without 63

Lime, on the virtues of carbonat of in cancer 496

———, phyſical characters and chemical characters of phoſphat and acid of 493

——— of oxalat of 495

——— of carbonat of 496

Lime-water, its uſe in the yellow fever 100

Lind, Dr. on typhus fevers 502

Lithotomy, on a caſe of 204

———, account of a new inſtrument for *(with a plate)* 394

Liver, extraordinary caſe of diſeaſed 129

Lobſtiere's, Cit. memoirs on the nutrition of the fœtus 255

London, account of diſeaſes in an eaſtern diſtrict of 16, 191, 275, 375, 469, 570

———, on the epidemical diſeaſe in 385

Low's, Mr. caſe of hydrothorax 417

——, method of taking caſts of the ear 551

Lucas's Mr. caſe of a ſingular extenuation in the limb 231

————, caſe of an uncommon affection in a limb 233

Macartney's, Mr. lectures announced 96

Madeira, introduction of vaccine inoculation at 310

M' Larty, Dr. on the yellow fever 34

Madreporit, analyſis of the 476

Magennis, Dr. on apoplexy 320

Mahon, Dr. on legal medicine with remarks 69

Mancheſter, account of the epidemic catarrhal fever at 522

Manganeſe, chemical analyſis of 476

Manſcey, Cit. on the diviſion of the ſymphiſis pubis 57

Marcet's, Dr. correſpondence with Dr. Jenner 462

Marcus, Dr. on the treatment of diſeaſes in the hoſpital at Bamberg 268

———, his method of treating intermittent fevers 304

Marſhall, Dr. introduces vaccine inoculation at Naples 436

Maurice, Mr. on the coincidence of diſeaſes 38

Maxillary ſinus, caſe of inflammation in the 423

Medical Dictionary, review of a new 285

———, lectures, ſee LECTURES

———, ſociety, liſt of the officers and council of the London 391

———, and phyſical intelligence 91, 192, 287, 338, 486, 581

———, and phyſical literature, critical retroſpect of 84, 177, 276, 377, 472, 571

Medicina Nautica, vol. III. reviewed 185

Medicine, obſervations on legal 69

————, account of a new one for the gout reviewed 286

Medicines, on the difficulty of aſcertaining the virtues of 19

Memminger, Dr. on fixed alkali in the hooping cough 176

Mental derangement, cautions concerning 72

Mercurial ointment, the internal adminiſtration of, recommended 487

Mercurial ſalts, new experiments on 9

Mercury, on the preparation of the mild ſublimate 81

————, on its application in malignant fever 98

Metal, account of a new 584

Miemit, chemical analyſis of the 476

Miller, Dr. on the yellow fever 105

——— in reply to Dr. Patterſon 302

Mitchill, Dr. on the opinions of 304

Modus operandi of opium, remarks on 153

Montpellier, program of prizes, propoſed by the ſociety at 91

Morbid Poiſons, obſervations on 309

Morley's Mr. obſervations on cataract 172

Mortification, on the uſe of muſk in 203

Moffman, Dr. on apoplexy 409

Moxas, on its uſe in loſs of ſpeech 389

Mulot, Cit. on the particular formation of a fœtus 461

Muſk, on its efficacy in gangrene and mortification 203

Naples, introduction of the vaccine inoculation at 436

Natron, chemical analyſis of the Egyptian and radiant 474

Natural hiſtory, French ſociety of profeſſors of 95

Neyronis, Cit. caſe of fatal pregnancy 470

Nicholas, Cit. chemical and medical experiments on the diabetes ſauar 12

North, letter to the hon. Frederic North, on vaccine inoculation 538

Nunn's, Mr. caſe of poiſon 247

Nutrition of the foetus, memoir on the 255

Obſervations on the epidemical diſeaſes now prevailing in London, &c. reviewed 385

———— on a late publication of Dr. Pearſon, entitled an examination of the report of the Houſe of Commons on the vaccine pock inoculation, reviewed 481

Obſervations on the conſtitution and diſeaſes of women, reviewed 279

———— on Dr. Pearſon's examination of the vaccine pock committee 286

Oeſophagus, deſcription of an inſtrument for extracting ſubſtances from the 6

———— caſe of contracted 552

Oil of walnut kernels, recommended in herpetic and cutaneous complaints 94

——, on purifying rape ſeed 582

Olive ore, chemical analyſis of the 475

Ophthalmia of Egypt, obſervations on the announced 196

Opium, obſervations and experiments on the modus operandi of 42, 335

———, Mr. Hill's remarks on Mr. Ward's theory of the modus operandi of 153

———, experiments relative to the chemical analyſis of 398

———, its uſe in febrile diſeaſes 269

———, its uſe in conſumption 270

Ovarian geſtation, curious caſe of 56

Oxalat of lime, chemical characters and phyſical properties of 495

Palladium, account of 585

Papaya, on the juice of the 79

Paralyſes, uſe of chenopodium ambroſiodes in 94

Paralyfes, on the application of Galvanifm in cafes of 136
——, on the effects of phofphorated ether in 300
Paris, account of the fociety of Natural Hiftory at 95
——, alarming diforder at 287
——, experiments by the Galvanic Society at 291, 487
——, report of the difeafe prevalent at 4 9
Paffion, extraordinary effects produced by 288
Patterfon, Dr. on the yellow fever 105
——, Dr. Miller's letter to 302
——, an American gentleman's letter on vaccine inoculation, to 196
——, Dr. Caldwell's letter to 581
Pearlftone, chemical analyfis of the Hungarian 476
Pearfon, Dr. communication from Dr. Heberden, to 95
——, Dr. obfervations on his examination of the report of the Vaccine Pock, Committee of the Houfe of Commons, by Thomas Creafer, reviewed 286
——, D . review of obfervations on the prefent epidemic catarrhal fever, or influenza, by 377
——, Dr. obfervations on a late publication of, by H. Hicks 481
Pennfylvania hofpital, account of the 250
Pepys, Mr. account of his Galvanic apparatus 390
Peripneumonia Notha, on the treatment of 376
Pharmacolith, chemical analyfis of the 476
Philadelphia, on the malignant fever at 97
——, account of the hofpital at 250
Phofphat of lime, phyfical characters and chemical properties of 493
——, difcovered in corn 583
Phofphorated ores, chemical analyfes of 475
Phosphoric acid, its benefits in hæmorrhagies 388
Phofphorus, on the medical effects of 299
Phofphuret of lime, preparation of 290
Phthifis, on the effects of digitalis and calomel in 263
Pitchftone, chemical analyfis of 475
Plague, on the propagation of the 502
Plants, on the fenfibility of 291
Plafter bandage, on its ufe in ulcers 113
Ploucquet's, Mr. experiments on the lungs 75
Poifon, a cafe of 247
Poifons, on morbid 309
Power's, Mr. obfervations on the ophthalmia of Egypt reviewed 575
Pregnancy, cafe of fatal 470
Premature delivery, cafe of 403
Pringle, Sir John, on the jail fever 592
Prize queftion of the fociety at Dijon 371
Prizes propofed by the fociety at Montpellier 91
Pulmonary confumption, benefits of opium in 270
Putrefaction of flefh in different kinds of gas, experiments on 219

Queftion on the fenfibility of plants 291
—— propofed by the fociety at Dijon 372
Queftions, prize, propofed by the medical fociety of Montpellier 91
Racchetti, Dr. account of a work by 437
Rafori, Dr. his Annali di Medicina, noticed 437
Reece's, Mr. Domeftic Medical Guide, reviewed 485
Rees, Dr. on the fea liver wort 24
Regnault, Dr. on the lichen iflandicus 20
Report on the difeafe called the gripe 419
—— of the medical board of Bombay 534
Refin, phyfical and chemical characters of animal bezoardic 498
Rheumatifm, the application of Galvanifm beneficial in 135
——, on the ufe of cold bathing in 549
Ricards's, Mr. reports of furgical cafes 13, 188, 272, 373, 467, 568
Rickets, benefits of the fpongia ufta in 487
Ring, Mr. on vaccination 64
——, letter to, from Dr. Friefe 128
——, on vaccine inoculation 296
——, his letter to Lord Hardwicke 541
——, his table fhewing the advantages of vaccine inoculation 543
Rogers's, Mr. cafe of cow-pox fuperfeding fmall-pox contagion 544
Ruffia, fuccefs of vaccine inoculation in 130
Ryan, Dr. on autumnal fever and contagion 213
Sacco, Dr. his exertions in Italy to promote vaccine inoculation 434
Salts, experiments on mercurial 9
Safsolin, chemical examination of the 474
Sawrey, Mr. on the venereal poifon 571
Scarlatina anginofa, prevalence of the 16
—— on the treatment of 511
—— improvement in the treatment of 249
Scarlet fever, on an epidemic 157
Schaeffer, Dr. on phthifis 21
Scheele ore, chemical analyfis of 473
Schwaz, Dr. his remedy againft obftinate cafes of tænia 290
Scorza, chemical examination of the 476
Scott, Mr. on cold applications in gout 547
Scrotal hernia, on ftrangulated 260
Sebacic acid, obfervations on the 78

Seguin's, Mr. remedy against intermitting fevers 567

Sheep, on the diseases of 455

Silesia, progress of vaccine inoculation at 129

Silex, composition of 496

Simmons's, Mr. W. observations on the use of the plaster-bandage in ulcers 113

———, on concussion of the brain 197

———, on a case of cataract 198

———, on the extirpation of the globe of the eye, *(with an engraving)* 200

———, on supposed dislocations 202

———, on musk in mortification 203

———, on a case of lithotomy, *(with an engraving)* 204

———, Mr. R. on the use of soda 267

Small-pox, on the extermination of the 356, 413

——— contagion, superseded by vaccine inoculation 544

——— hospital, report of the committee of the 194

Smith's case of contracted œsophagus 552

Soda, on the use of 267

——— on the uriat of 492

Somerset, account of the epidemic in the county of 505

Sore throat, observations on the 157

Speech, use of moxas in cases of the loss of 389

Spongia usta, its efficacy in glandular diseases 486

Steatomatous tumour, case of, *(with a plate)* 17

Stimulants, observations on the term 344

Stones, on urinary 242

Strenger, Mr. on the Galvanic influence in deafness 290

Strictures of the urethra, on the treatment of 188

——— account of an instrument for removing 4

Struma, efficacy of spongia usta in 486

Sublimate mercury, on the preparation of mild 81

Sue's history of Galvanism, a translation of, announced 196

Suppuration, case of thoracic 426

Surgery, observations on select subjects in *(with a plate)* 197

Surgery, review of Bell's principles of 14, 177, 276

Surgical instruments, descriptions of several new *(with a plate)* 1

Surgical cases in Finsbury Dispensary, monthly report of, 13, 188, 272, 373, 467, 568

Swallowing, observations on difficulty of 293

Syers's, Mr. lectures announced 96

Symphisis pubis, on the division of the 57

Table, shewing the advantages of vaccine inoculation 543

Tænia, remedy against obstinate cases of 290

Tar-water, its use in venereal complaints 581

Taynton, Mr. on cold applications in gout 547

Tellurium, chemical examination of 473

Terras, M. on the internal administration of mercurial ointment 487

Thenars, Cit. on the sebacic acid 78

———, on purifying rapeseed oil 582

Thigh, case of the amputation of the 499

Thomas's, Mr. Lectures announced 196

Thoracic adhesions, case of 359

——— suppuration, case of 426

Tinea capitis, case of 60

Tomlinson, Mr. on the influenza 530

Tongue, account of a new instrument for the 7

Tourniquet, account of a new one for the tongue *(with an engraving)* 7

Trephine, on the use of the 273

Trotter's, Dr. Medicina Nautica, vol. iii. reviewed 185, 282

——— reply to Mr. Henry 448

Tumour, case of steatomatous *(with an engraving)* 17

Tupper's, Mr. experiments on Galvanic contractions 146, 235

Typhus, on the affusion of cold water in 206

———, how treated in the hospital at Bamberg 268

———, Dr. Lind's Letter on 502

Ulcers, on the use of the plaster-bandage in 113

———, how treated in the hospital at Bamberg 271

Umbilico-mesenteric vessels, observations on the 170

Urat of ammoniac, characters of 492

Urethra, account of an instrument for removing strictures of the 4

———, observations on strictures of the 188

Uriat of soda, characters of 492

Uric acid, characters of the 491

Urinary stones and calculi in men, observations on the 242

Urine, observations on the 349

Vaccination, Mr. Ring on 64

———, Mr. Wagstaffe on 361

———, Mr. Dennett on 363

———, Mr. Washbourn on 370

Vaccine inoculation, Mr. Maurice on 39

———, report of the Small-pox hospital on 194

———, success of, in America 196

———, its introduction into the island of Madeira by Dr. Adams 309

———, Capt. Keyt's letter relative to 391

———, letter from Dr. De Carro to Dr. Jenner on 450

———, letter from Mr. Christie to Sir Walter Farquhar on 457

Vaccine inoculation, correspondence between Drs. Marcet and Jenner on 462

——, Cit. Goetz on the inutility and danger of, reviewed 484

——, Mr. Wagstaffe's case of 533

——, report of the medical board on 534

——, Lord Hardwicke's letter on 540

——, Dr. Jenner and Mr. Ring's answer to ditto 541

——, table shewing the advantages of 443

——, Mr. Rogers's case of 544

Vaccinator, Mr. Ring's description of a 297

Vaccine matter, Mr. Giraud's mode of preserving *(with a drawing)* 397

——, Dr. De Carro's observations on 451

——, Dr. Jenner's observations on 463

——, Dr. Marcet's mode of preserving 464

——, how to transmit in a dry state 541

Van Mons, his mode of preparing the aqua laurocerasi 288

Varicella, case of vaccination suspended by 370

Vauquelin on the juice of the papaya 79

——, on phosphat of lime 584

——'s analysis of the boracites 92

Venereal disease, how treated in the hospital at Bamberg 271

——, internal administration of mercurial ointment recommended in the 487

——, tar-water recommended in 581

—— poison, enquiry into the effects of 571

Vinegar, aromatic, Mr. Henry's claim to the discovery of 307

——, Dr. Trotter's letter concerning 448

Vitriolated zinc, on the use of 55

Wadd, Mr. on cold water in the gout 205

Wadley, Mr. on the influenza 515

Wagstaffe's, Mr. Lectures announced 96, 292

——, case of vaccination 361

——, case of thoracic adhesions 359

——, case of vaccine 532

Walker's, Dr. John, vindication of the conduct of government towards him 62

——, Dr. S. observations on the constitution and diseases of women, reviewed 279

Walnut kernel, virtues of the oil of 94

Ward, Mr. on alcohol, &c. 40

—— Mr. on the modus operandi of opium 335

—— Mr. his case of elephantiasis 545

Wastell's, Mr. case of thoracic suppuration 427

Water cold, on the virtues of in gout 547

—— in typhus 207

—— in rheumatism 549

Wichmann's Dr. observations on dysphagia 293

Wilkinson's, Mr. work on the willow bark announced 392

——, history of Galvanism announced 488

Willan, Dr. letter from Dr. Adams to 309

Women, observations on the constitution of 279

Woodforde, Dr. on the influenza 505

Wound, case of gun-shot 165

Yellow fever, Dr. Harris on the 25
——, Dr. Mac Larty on the 34
——, Dr. Burke on the 37
——, Dr. Currie's observations on the treatment of the 97
——, Dr. Miller on the 103, 302
——, Dr. Patterson on the 105
——, method of treating 581

Zinc, the acetite of recommended in gonorrhœa 53
——, use of the vitriolated 55
Zirkon, chemical analysis of the 476
Zufera, account of the 562

### REFERENCES TO THE PLATES.

Dr. Clarke's newly-invented Surgical Instruments — — 1
Mr. Simmons's Case of Lithotomy — — — — — 200
—— Extirpation of the Globe of the Eye — *ib.*
Mr. Abernethy's Gorget — — — — — — — 393
A. F. Fourcroy's Representations of the Materials which compose Calculi, &c. — — — — — — — — 489

